# Case report: A case series report: recurrent urolithiasis as a risk factor for upper tract urothelial carcinoma

**DOI:** 10.3389/fonc.2026.1850083

**Published:** 2026-07-01

**Authors:** Yuke Zhang, Shuxian Shen, Weiyuan Li, Yangyang Hu, Yu Zhang, Keqiang Li, Guangchun Wang

**Affiliations:** 1 Department of Urology, Shanghai Tenth People’s Hospital, Tongji University, Shanghai, China; 2 Cancer Institute, School of Medicine, Tongji University, Shanghai, China; 3 Department of Nephrology, Nantong Tongzhou District People’s Hospital, Nantong, China; 4 Department of Urology, Peking University First Hospital, Beijing, China; 5 Department of Urology, People’s Hospital of Shigatse City, Shigatse, China

**Keywords:** case report, stone, upper urinary tract carcinoma, urolithiasis, UTUC

## Abstract

**Introduction:**

Urothelial carcinoma of the bladder (UBC) and upper tract urothelial carcinoma (UTUC) are distinct malignancies with differing prognoses and molecular characteristics despite their shared histological origin. Emerging evidence suggests that chronic inflammation, often driven by specific carcinogenic exposures, plays a critical role in tumour development. While the oncogenic role of various chemical agents in UTUC is well established, the potential contribution of recurrent urolithiasis remains underappreciated and insufficiently studied. Given the frequent observation of inflammatory polyps surrounding upper tract calculi, further investigation into this association is warranted.

**Case presentation:**

This case series reports on three patients with UTUC in the context of recurrent urolithiasis. Case 1 involves a 53-year-old male patient with a history of repeated ureteric stones and inflammatory polyps who was later diagnosed with low-grade invasive urothelial carcinoma. Case 2 describes a 46-year-old female patient with bilateral renal calculi who developed high-grade invasive UTUC following long-standing calculus disease. Case 3 is a 57-year-old male patient who had recently ingested herbal medicine containing aristolochic acid and was found to have high-grade UTUC, with concurrent urolithiasis potentially contributing to carcinogenesis. In each instance, urothelial carcinoma was initially concealed by polypoid lesions surrounding impacted stones, highlighting the associated diagnostic challenges.

**Conclusion:**

This case series underscores the potential oncogenic role of chronic inflammation induced by recurrent upper urinary tract calculi. Persistent stone-related mucosal injury may contribute to UTUC pathogenesis. Clinicians should maintain a high index of suspicion and perform targeted biopsies of polypoid lesions encountered during the endoscopic management of urolithiasis. Enhanced awareness may facilitate earlier detection and intervention, particularly in patients with recurrent calculus disease.

## 1 Introduction

Urothelial carcinoma of the bladder (UBC) and upper tract urothelial carcinoma (UTUC), which includes tumours of the renal pelvis and ureter, are both classified as urothelial carcinomas (1). Growing evidence suggests that these malignancies are not merely distinguished by anatomical location; rather, they differ significantly in prognosis and molecular characteristics (2–4). It is recognised that UTUC is associated with a considerably poorer prognosis than UBC (1, 5).

Distinct tumour subtypes often exhibit unique molecular features that are closely linked to specific carcinogenic factors. These carcinogenic factors do not simply initiate tumour development; rather, they fundamentally determine the tumour’s genomic and biological profile through distinctive molecular mechanisms (6, 7). For instance, in lung cancer, chemical compounds in tobacco smoke leave characteristic “smoking mutational signatures”. These compounds are primarily associated with small cell lung cancer and squamous cell carcinoma, whereas non-smokers more frequently develop adenocarcinoma (8, 9). Similarly, in hepatocellular carcinoma, differences exist between tumours caused by hepatitis B and C viruses and those associated with aflatoxin exposure, with each corresponding to distinct molecular subtypes (10, 11). Consequently, identifying the unique carcinogenic factors that drive each tumour subtype is critical for early intervention and precision therapy.

Although epidemiological studies have shown that both UTUC and UBC are closely associated with chronic inflammation, the risk factors contributing to this inflammation differ markedly between UTUC and UBC (5, 12, 13). One such distinction lies in the role of urolithiasis. Upper urinary tract stones are more likely to cause obstruction and mucosal injury, often resulting in a more pronounced inflammatory response. In contrast, bladder stones typically provoke milder inflammation, manifesting primarily as irritative lower urinary tract symptoms. Recurrent upper tract stones can exert persistent irritation on the urothelium, leading to chronic inflammation.

At the cellular level, a key consequence of chronic inflammation—particularly when accompanied by bacterial infection on the stone surface—is the excessive generation of reactive oxygen species (ROS) by activated inflammatory cells. The resultant oxidative stress can induce direct oxidative DNA damage, creating a pro-mutagenic environment. If these ROS-induced DNA lesions are not efficiently repaired by intrinsic DNA repair mechanisms, they can lead to stable genetic mutations and, potentially, inflammation-driven carcinogenesis (14–16). This mechanism is well documented in other malignancies, such as the progression from chronic gastritis to gastric cancer and from cholangitis to cholangiocarcinoma (17, 18).

This case series presents three patients diagnosed with UTUC in the context of urolithiasis, aiming to enhance clinicians’ awareness of the potential oncogenic risk posed by recurrent upper urinary tract stones.

## 2 Case presentation

### 2.1 Case 1

A 53-year-old male patient with no history of smoking or alcohol abuse presented with a medical history of hypertension and recurrent urolithiasis, and no family history of hereditary diseases. The patient was employed as a long-distance lorry driver, a role involving prolonged periods of sedentary activity. In 2015, he experienced severe renal colic caused by a right ureteric calculus and underwent lithotripsy at a local hospital. He subsequently received treatment on several occasions for right renal pelvic and ureteric calculi in January 2017, January 2018, and August 2018, each time due to recurrent pain.

**Figure 1:**
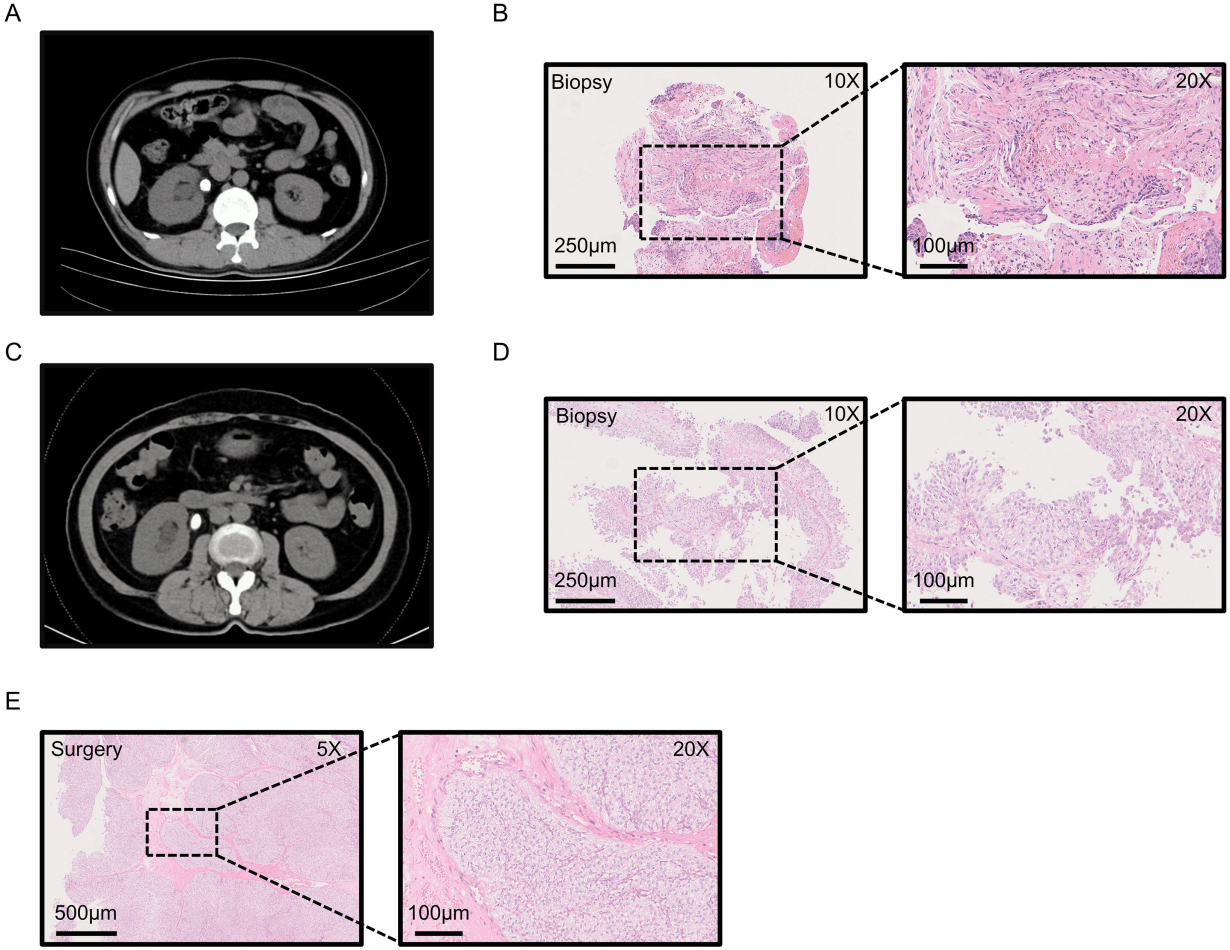
Urolithiasis-associated UTUC: case 1. **(A)** Initial CT imaging of the patient showing a ureteropelvic junction stone at the patient’s first clinical visit. **(B)** Initial intraoperative tissue biopsy stained with H&E, revealing inflammatory polypoid tissue without evidence of malignancy. **(C)** Follow-up CT imaging demonstrating recurrent stone formation at the right ureteropelvic junction. **(D)** Intraoperative tissue biopsy stained with H&E showing one specimen consistent with low-grade urothelial carcinoma. **(E)** Postoperative tumour tissue stained with H&E.

In May 2019, the patient presented with recurrent aching discomfort in the lumbar region. An ultrasound scan performed at a local hospital revealed a ureteric stone with marked hydronephrosis. He was subsequently referred to our institution for further management. On admission, laboratory tests showed a white blood cell count of 13.1 × 10^9^/L, with 79.5% neutrophils and a procalcitonin level of 0.07 ng/mL. Urinalysis revealed 6 red blood cells/μL. Abdominal CT imaging indicated moderate hydronephrosis, with a calculus measuring approximately 12 × 11 × 8 mm located at the ureteropelvic junction (**Figure 1A**).

During ureteroscopic lithotripsy, marked narrowing of the upper ureteric lumen was observed, along with extensive polypoid growths surrounding the stone. A biopsy was obtained from the impacted polypoid tissue. Histopathological examination revealed inflammatory polyps (**Figure 1B**). The patient made an uneventful postoperative recovery. Follow-up ultrasonography was unremarkable, and he was subsequently discharged.

In February 2020, routine ultrasonography performed during a general health check again showed hydronephrosis in the upper ureter, prompting the patient to return to our hospital. The patient reported no significant symptoms. Based on further CT imaging findings, he was admitted with a presumed diagnosis of ureteric calculi (**Figure 1C**). On admission, his white blood cell count was elevated at 11.2 × 10^9^/L, with 78.5% neutrophils and a procalcitonin level of 1.48 ng/mL. Urinalysis revealed 101 red blood cells/μL. Given his history of recurrent urolithiasis, parathyroid hormone levels were assessed and found to be within the normal range.

Another session of transurethral ureteroscopic holmium laser lithotripsy was performed. Intraoperatively, extensive polypoid tissue was again observed surrounding the impacted stone. Biopsies were obtained from the polypoid lesions; however, histopathological examination of one of the three biopsy specimens revealed mildly atypical urothelial cells with a papillary architecture, consistent with low-grade papillary urothelial carcinoma (**Figure 1D**). The following month, the patient underwent laparoscopic nephroureterectomy. Postoperative histopathology confirmed a diagnosis of low-grade invasive urothelial carcinoma (T1N0M0), with no evidence of lymph node involvement (**Figure 1E**). The patient subsequently received intravesical instillations of epirubicin for 6 months. At the time of writing, he is recovering well, with no signs of recurrence.

### 2.2 Case 2

A 46-year-old female patient with a history of recurrent urolithiasis presented to our hospital. She had no history of smoking, alcohol abuse, or hereditary disease. The patient was first diagnosed with bilateral renal calculi 10 years previously and had undergone ureteroscopic lithotripsy at another hospital in 2019. After the first postoperative year, she experienced multiple episodes of lumbar pain but declined further inpatient assessment and treatment. The patient opted for conservative management at home, including increased fluid intake and physical activity such as skipping. This approach resulted in the spontaneous passage of the stones and temporary relief of symptoms.

**Figure 2:**
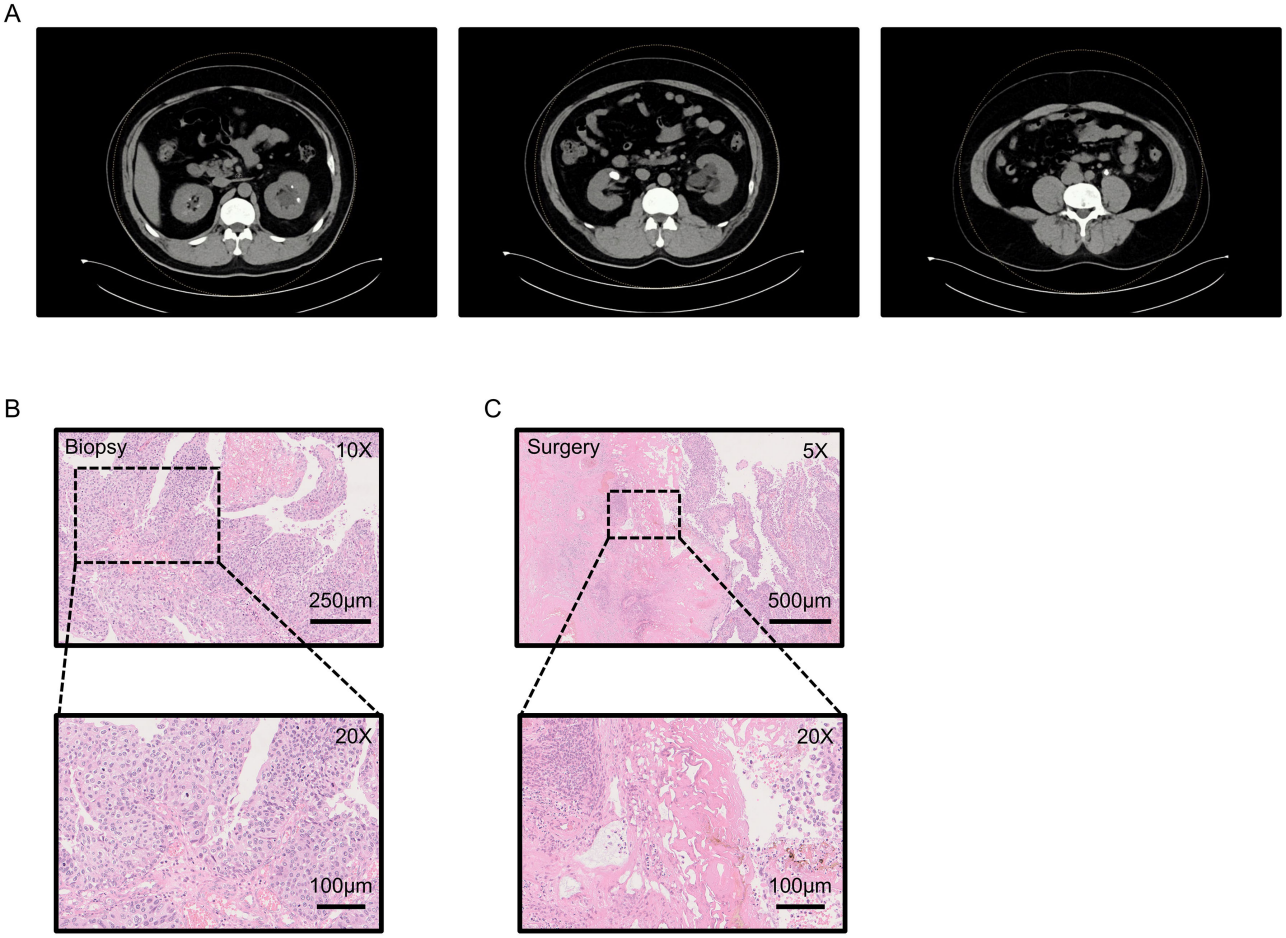
Urolithiasis-associated UTUC: case 2. **(A)** CT imaging showing multiple bilateral renal calculi, severe hydronephrosis of the left kidney, and a left ureteropelvic junction stone. **(B)** Intraoperative tissue biopsy stained with H&E, revealing one specimen consistent with high-grade invasive urothelial carcinoma. **(C)** Postoperative tumour tissue stained with H&E.

In April 2024, the patient again developed lumbar pain, which did not resolve after one week of self-administered conservative treatment. She subsequently presented to our hospital for further evaluation. On admission, laboratory investigations revealed a white blood cell count of 11.6 × 10^9^/L, with 73.5% neutrophils. Renal function tests showed a serum creatinine level of 50.6 μmol/L and a serum potassium level of 3.65 mmol/L. Urinalysis revealed 168 red blood cells/μL. Abdominal CT imaging demonstrated multiple bilateral renal calculi, severe hydronephrosis of the left kidney, and a left ureteric stone. A calculus measuring 8 × 6 × 10 mm was identified in the upper left ureter (**Figure 2A**).

The patient underwent holmium laser lithotripsy via flexible ureteroscopy. The impacted stone was fragmented and dislodged from the adherent polypoid tissue, then flushed back into the renal pelvis for staged management. No residual stones were observed at the site of impaction. Multiple biopsies were obtained from the polypoid tissue for histopathological analysis.

Two weeks later, histology revealed high-grade urothelial carcinoma (**Figure 2B**). The planned second-stage lithotripsy was therefore cancelled, and surgical management was revised to laparoscopic nephroureterectomy. Abdominal CT and chest CT scans showed no abnormalities. Postoperative histopathology reconfirmed the diagnosis of high-grade invasive urothelial carcinoma (T2N0M0) (**Figure 2C**).

The patient was discharged in a stable condition two weeks postoperatively. In accordance with her wishes and those of her family, she sought further treatment at a hospital near her place of residence. Attempts were made to contact the patient by telephone; however, she was lost to follow-up after discharge, and her subsequent clinical outcomes remain unknown.

### 2.3 Case 3

A 61-year-old male patient presented to our hospital with a 2-month history of discomfort in the right lumbar region, which had worsened over the preceding two days. He had no history of smoking or alcohol abuse and denied any family history of hereditary disease. Eight months previously, he had experienced an episode of acute cerebral infarction, from which he recovered following treatment. His condition remained stable at the time of presentation.

**Figure 3:**
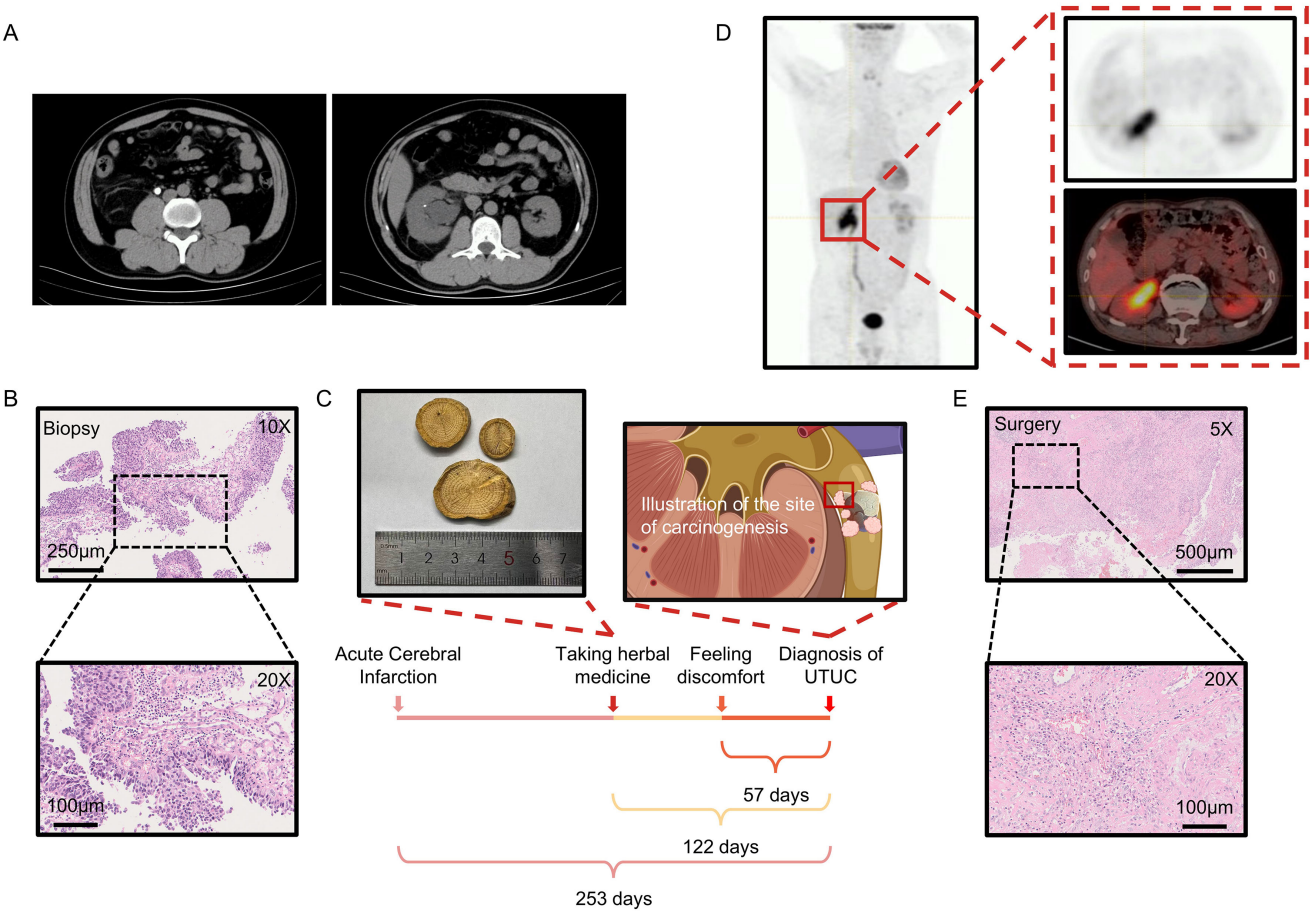
Urolithiasis-associated UTUC: case 3. **(A)** CT imaging showing a right ureteropelvic junction stone and associated hydronephrosis of the right kidney. **(B)** Intraoperative tissue biopsy stained with H&E, revealing one specimen consistent with high-grade urothelial carcinoma. **(C)** Timeline of the patient’s clinical course. **(D)** PET-CT imaging showing no evidence of distant metastasis. **(E)** Postoperative tumour tissue stained with H&E.

On admission, laboratory tests revealed a white blood cell count of 5.99 × 10^9^/L, with neutrophils accounting for 66.7%, and a procalcitonin level of 0.03 ng/mL. Urinalysis revealed 5 red blood cells/μL. Abdominal CT imaging demonstrated right-sided hydronephrosis and a right ureteric calculus measuring 7 × 6 × 11 mm (**Figure 3A**).

Ureteroscopic intervention revealed polypoid lesions, both proximal to and distal to the impacted stone. Histopathological analysis of the biopsy specimens obtained intraoperatively revealed that the polypoid lesion located distal to the stone exhibited marked cytological atypia, conspicuous loss of cellular polarity, enlarged hyperchromatic nuclei, and an increased nuclear-to-cytoplasmic ratio, findings consistent with high-grade urothelial carcinoma (**Figure 3B**).

Subsequent multidisciplinary team (MDT) discussion included a detailed review of the patient’s medical history. It was noted that, following his stroke, the patient had been taking a Chinese herbal preparation containing Aristolochia manshuriensis (commonly known as “mutong”) (**Figure 3C**). Given the established association between aristolochic acid exposure and UTUC, together with the known high concentration of aristolochic acid in Aristolochia species, a sample of the herbal preparation was submitted for toxicological analysis. The results confirmed the presence of a high concentration of aristolochic acid in the formulation.

In December 2024, a positron emission tomography-computed tomography (PET-CT) revealed no evidence of distant metastasis (**Figure 3D**). Later that month, the patient underwent radical nephroureterectomy. Postoperative pathology confirmed high-grade urothelial carcinoma (T2N1M0) with involvement of a single lymph node (**Figure 3E**).

The patient subsequently received four cycles of chemotherapy with gemcitabine plus cisplatin. At the time of writing, the patient is recovering well, with no clinical or radiological evidence of disease recurrence.

## 3 Discussion

Distinct molecular characteristics are often observed across different subtypes of the same tumour, and these subtype-specific molecular profiles are frequently shaped by unique carcinogenic factors. As such, elucidating the risk factors associated with each tumour subtype is critical for both early intervention and the development of precision therapies.

In the upper urinary tract, recurrent urolithiasis is a common cause of chronic inflammation. Urological surgeons frequently encounter inflammatory polyps surrounding renal pelvic and ureteric calculi. However, unlike aristolochic acid, which has a well-established causal association with UTUC, the role of recurrent calculi in UTUC pathogenesis remains controversial. Among major clinical guidelines, only those of the Chinese Urological Association (CUA) have acknowledged a potential link between UTUC and chronic inflammation induced by urolithiasis. In contrast, other authoritative guidelines, including those of the European Association of Urology (EAU) and the American Urological Association (AUA), maintain that there is currently insufficient evidence to support this association.

Several factors contribute to this lack of consensus. First, UTUC has only been recognised as a distinct subtype of urothelial carcinoma within the past two decades. Second, it is inherently difficult to generate robust epidemiological data on chronic inflammation caused by recurrent stones.

Our own experience illustrates these challenges. Obtaining a sufficient number of cases for robust statistical analysis proved challenging. A retrospective review of clinical records from the two years preceding UTUC diagnosis identified a history of recurrent urolithiasis in a subset of patients. However, the number of cases identified (11 cases of recurrent urolithiasis out of 739 patients with UTUC) was limited due to several factors, including the low incidence of UTUC, the fact that recurrent urolithiasis is only one of many risk factors, and the possibility of recall bias.

Furthermore, establishing a clear causal relationship between UTUC and calculi is challenging, and identification of such cases is often opportunistic rather than systematic. For example, in Case 1, the initial biopsy of a polyp surrounding the stone revealed only inflammatory changes, whereas a second biopsy performed within the year confirmed urothelial carcinoma. Whether this discrepancy reflects disease progression or sampling error is difficult to ascertain. Compounding this issue is the fact that, during stone fragmentation procedures, urologists often forego biopsies of adjacent polyps due to concerns regarding sepsis, thereby potentially missing diagnostic opportunities.

Additional confounding factors further obscure causality. For instance, in our pathological data collection, one patient was found to have a malignant lesion within a polyp adjacent to a stone, but also had a long-standing history of smoking. In this case, it was not possible to determine whether the carcinogenic risk was primarily attributable to the stone or to tobacco exposure.

Case 3 also initially appeared to involve a confounding factor, as the patient had a documented history of aristolochic acid ingestion. However, previous studies suggest that aristolochic acid–associated carcinogenesis typically requires prolonged exposure, whereas this patient had been exposed for less than 3 months. We therefore hypothesised that the coexisting stone disease may have potentiated the carcinogenic effects of aristolochic acid, possibly through the induction of hydronephrosis and prolonged local exposure. This could explain why such a short duration of aristolochic acid intake was associated with the development of UTUC in this patient.

## 4 Conclusion

This case series provides further evidence supporting an association between urolithiasis and UTUC. Despite the challenges in establishing definitive, evidence-based guidelines and the time required to build a broader consensus, urologists should remain vigilant regarding the potential association between urinary tract stones and UTUC. Special attention should be paid to performing biopsies of polypoid lesions associated with stones, as these are often overlooked during routine procedures.

## Data Availability

The original contributions presented in the study are included in the article/Supplementary Material. Further inquiries can be directed to the corresponding authors.
